# From Pornography Consumption to Sexually Violent Practices: Uncovering the Hidden Influence of Sexual Norms

**DOI:** 10.3390/bs15030243

**Published:** 2025-02-20

**Authors:** Carmen M. Leon, Tatiana Quiñonez-Toral, Eva Aizpurua

**Affiliations:** 1 School of Law, University of Castilla-La Mancha, 02071 Albacete, Spain; tatiana.quinonez@uclm.es; 2 Centre for Social Survey Transformation, National Centre for Social Research, London EC1V 0AX, UK; eva.aizpu@gmail.com

**Keywords:** sexual violence, pornography consumption, sexually permissive attitudes, sexual double standard, gender differences

## Abstract

Understanding the dynamics of sexually violent behavior is essential for developing effective interventions and policies that promote safe and respectful sexual relationships. An important area of research in this context is examining the influence of pornography on sexual behavior, which can inform these efforts. This study explores how sexually permissive attitudes and acceptance of the sexual double standard mediate the relationship between pornography consumption and engagement in violent sexual practices among a sample of the general population in Castilla-La Mancha, Spain (N = 1003; 50.7% men). The sexual double standard refers to the societal belief that men and women should be judged differently for the same sexual behaviors, with men often praised or excused for promiscuity, while women face stigma and shame. The findings reveal gender differences in engagement in sexually violent practices during sexual relationships. Men are more likely to perpetrate these behaviors, while women are more likely to experience them. Importantly, both lifetime and last year pornography consumption are associated with an increased involvement in such practices. This relationship is partially mediated by sexually permissive attitudes, with a stronger mediation effect observed in men. Practitioners can leverage these insights to develop comprehensive strategies that mitigate the risks associated with pornography consumption.

## 1. Introduction

Extensive research has examined the impact of pornography on sexual behavior, often focusing on its connection to attitudes that support violence against women, such as rape myth acceptance, sexual objectification, and adversarial sexual beliefs (Allen et al., 1995; Hald et al., 2010; Hedrick, 2021). However, findings regarding its link to sexual aggression have been mixed. For example, Wright et al. (2016) identified a correlation between pornography consumption and acts of physical and verbal sexual aggression, while Ferguson and Hartley (2022) found no link between pornography consumption and the perpetration of sexual aggression across various study designs.

In Spain, where this study was conducted, pornography consumption is notably high. According to Pornhub’s (2024) annual report, Spain ranks 11th globally for pornography consumption, with 60% of individuals aged 16 to 29 reporting usage (Gomez-Miguel et al., 2023). However, Pornhub data reflect activity on a single platform, and do not capture consumption across other popular sites. As such, it is unclear whether Pornhub traffic accurately represents global pornography consumption, as different platforms may dominate in other regions. While comprehensive data on adult consumption are lacking, Ballester-Arnal et al. (2023) found that over 85% of Spaniards aged 26 and older have consumed pornography at some point. This high prevalence, alongside rising rates of both formal reports and self-reported cases of sexual aggression (Eurostat, 2022; Ministry of the Interior, 2023), prompted the Spanish government to focus on restricting minors’ access to online pornography. In 2024, the government announced the introduction of an age-verification tool designed to prevent minors from accessing pornographic content. The proposal has been well-received by the public, as it aims to protect minors from various online risks, including gambling, image manipulation, and harassment. Originally scheduled for implementation in mid-2024, the initiative has been postponed, due to complex negotiations with pornographic websites and challenges related to its implementation.

Recent surveys characterize Spain as having an egalitarian and sexually permissive approach to sexuality. The survey on Perceptions towards Gender Equality and Gender-Based Stereotypes (Centro de Investigaciones Sociológicas, 2024) reports that 7 out of 10 Spanish residents believe that “taking the initiative in a sexual relationship” is equally characteristic of both men and women. Similarly, the survey on Post-Pandemic Social and Affective Relationships (Centro de Investigaciones Sociológicas, 2023) found that 69% of respondents agree that “it is acceptable to have sex with someone without loving them”.

Despite evidence suggesting that pornography influences sexual behavior, there is a notable lack of research examining its connection to sexual attitudes and behaviors in Spain. Existing studies have primarily focused on indicators of sexual aggression, such as aggressive sexual fantasies and rape myth acceptance, male rape proclivity (Goodson et al., 2020; Palermo et al., 2019), and pornography consumption among convicted sexual aggressors (Loutzenhiser et al., 2024). Only a few studies (Ezzell et al., 2020; Merlyn et al., 2020) have investigated the relationship between pornography and violent sexual practices often depicted and normalized in pornography, such as spanking, gagging, hair-pulling, and slapping. However, previous research has not disaggregated the analysis based on whether individuals have perpetrated or experienced such behaviors (Merlyn et al., 2020), nor has it specifically focused on examining how these practices relate to sexual norms. Instead, it has primarily focused on whether individuals enjoy these violent behaviors (Ezzell et al., 2020).

Although some studies do not specify the type of pornography consumed, the majority focus on mainstream content, which is predominantly heteronormative and tailored to a heterosexual audience (e.g., MILF, Latina, stepmom genres). As a result, our literature review primarily addresses heteronormative pornography, and does not encompass the full spectrum of pornographic material targeted at the LGBTQ+ community. This is an important distinction, as the consumption patterns and potential effects of pornography may vary across different genres.

Addressing this topic is crucial for several reasons. Understanding the relationship between pornography consumption and violent sexual practices provides insight into how these behaviors are normalized and potentially perpetuated in society. While prior research has extensively focused on sexual aggression and rape, there remains a gap in understanding how exposure to pornography might influence everyday behaviors, particularly regarding consent, aggression, and power dynamics in intimate relationships. This knowledge is essential for developing effective interventions and educational programs that promote healthier, consensual sexual practices. Additionally, it can inform policy decisions regarding the regulation of pornographic content and its accessibility.

Building on the 3AM Theory (Wright, 2011), this research investigates the potential relationship between pornography consumption and the occurrence of violent practices within sexual relationships. Specifically, it addresses two primary research questions: (1) What is the relationship between pornography consumption and the occurrence of violent sexual practices in sexual relationships? (2) If such a relationship exists, to what extent do sexual permissiveness and the sexual double standard mediate this association? By exploring these mediating factors, this research provides a more nuanced understanding of how pornography consumption may influence engagement in sexually violent behaviors.

### 1.1. Theoretical Framework

The sexual script approach has been widely used in the literature to examine the relationship between pornography consumption and the endorsement of specific sexual attitudes and behaviors (Wright et al., 2022). The 3AM Theory (Wright, 2011) provides further insight into this relationship by suggesting that individuals acquire new behavioral scripts through media exposure (Acquisition). These scripts can later be triggered by similar media content (Activation) and applied in real-life situations (Application). According to this model, pornography provides consumers with sexual scripts where violence is depicted as normal, acceptable, and even enjoyable. The activation of such scripts is influenced by content-related factors (e.g., attractiveness), individual characteristics (e.g., gender, motivations), situational factors (e.g., sexual arousal), and the accessibility of the script (e.g., vividness). The application of these scripts is further influenced by beliefs and moral standards. If a script conflicts with internal moral values, it may be suppressed (Wright, 2011).

### 1.2. Pornography Consumption and Violent Sexual Behavior

As noted, the relationship between pornography consumption and violent sexual behavior has yielded mixed findings (Ferguson & Hartley, 2022; Wright et al., 2016). However, the potential impact of pornography on sexual behavior should not be underestimated. A 2010 study found that 88% of pornographic scenes featured physical violence, and 49% included verbal violence (Bridges et al., 2010). In heterosexual mainstream pornography, including those labeled as feminist, violence is predominantly male and directed toward women (Fritz & Paul, 2017; Whisnant, 2016). Commonly depicted acts of violence include spanking, gagging, slapping, and choking (Bridges et al., 2010), with explicit verbal consent and condom use rarely represented (Grudzen et al., 2009; Jozkowski et al., 2019).

Mainstream pornography often portrays sexual violence as pleasurable, eroticizing women’s explicit resistance to certain practices or rape (Alario, 2021; Jeffreys, 2022). This trivializes the word “no” and promotes sexual scripts that normalize violence and “token resistance” (Foubert et al., 2019). Several studies have explored the relationship between pornography consumption and violent, degrading, or risky behaviors. For example, Merlyn et al. (2020) found that pornography users were more likely than non-users to engage in behaviors such as hair-pulling, hitting, spanking, face-slapping, or choking their sexual partners without consent. More recently, Wright et al. (2023) found that pornography consumption predicted an increased likelihood of choking a sexual partner among heterosexual college men.

The normalization of violent sexual behaviors in widely consumed media, such as pornography, contributes to the justification of violence during sexual encounters, shaping societal views on acceptable sexual practices. Although these behaviors have become largely normalized within conventional sexual encounters, they remain inherently violent and pose risks. For instance, sexual choking, which is widely normalized in pornography and increasingly common in sexual relationships (Herbenick et al., 2021), can cause severe pain and lasting effects (Jeffreys, 2022). In extreme cases, it can lead to fatal outcomes, such as death by asphyxiation (Sendler, 2018). The emotional consequences of these practices also warrant attention. The portrayal of violent sexual behaviors as pleasurable can create confusion when individuals do not find them enjoyable in practice. Ezzell et al. (2020) observed that while most women do not enjoy degrading practices, the majority of men do. This discrepancy can lead to dissatisfaction and psychological distress.

A critical concern is the thin line between consent and coercion in the normalization of violent sexual practices. When individuals internalize these behaviors as normal and pleasurable, they might overlook the need for consent or disregard the desires of their partners. For example, Fahs and Gonzalez (2014) found that many women engage in anal sex despite intense physical pain or discomfort, to satisfy their partner’s desires or because they perceive it as a normal part of sexual relationships. This normalization also affects those who perpetrate these practices. A recent study found that more men than women choked their sexual partners during intercourse, believing it to be safe, pleasurable, and not requiring explicit consent (Wright et al., 2023). The normalization of violent sexual practices poses important risks to both physical and emotional well-being, especially for women, highlighting the need for greater awareness and critical examination of these behaviors.

### 1.3. Pornography Consumption, Sexual Permissiveness, and Sexual Behavior

Sexual permissiveness refers to an attitude or belief system that supports a broad range of sexual behaviors and expressions without strict moral or societal constraints. It reflects a liberal perspective on sexual activities, where behaviors that some might disapprove of are accepted or normalized (Laner et al., 1978). Research has demonstrated a correlation between pornography consumption and more permissive sexual attitudes (Tokunaga et al., 2019; Wright, 2024). For example, Wright (2013b), using data from the United States General Social Survey (1973–2010), found that individuals who had consumed pornography in the preceding year exhibited more favorable attitudes towards premarital, teenage, and extramarital sex. Longitudinal studies have also challenged the Selective Exposure Theory, which posits that individuals prefer media aligned with their pre-existing attitudes, by showing that pornography consumption at Time 1 predicts increased sexual permissiveness at Time 2, even after controlling for initial levels of sexual permissiveness (Leonhardt & Willoughby, 2018; Wright, 2013a). This evidence suggests that pornography consumption fosters sexual permissiveness, rather than merely reflecting it.

These findings are unsurprising, given that mainstream pornography often portrays impersonal and emotionally detached sexual encounters, framing sex as casual, purely physical activity (Peter & Valkenburg, 2006; Tokunaga et al., 2019). As Miller and McBain (2021) noted, “pornography is much more likely to depict casual sex than sex within the context of a long-term relationship” (p. 31). Indeed, Rasmussen et al. (2019) found that committed relationships appeared in only 7.9% of mainstream pornographic videos, while Seida and Shor (2021) reported that affectionate behaviors such as kissing, caressing, or sweet-talking were present in only 1.9% of heterosexual pornographic videos. Consequently, pornography often conveys scripts where sexual interactions are casual, impersonal, and laden with violence, sexual objectification, and rigid gender stereotypes (Criado-Pajuelo, 2022; Willis et al., 2022). As Rasmussen et al. (2019) observed, “consumers might come to see casual sex as normative, exciting, or desirable” (p. 580).

An impersonal approach to sexuality has been central to predicting sexually violent behavior. According to the Confluence Mediational Model of Sexual Aggression (Malamuth, 1986; Malamuth & Hald, 2017), individuals who view sex as impersonal may see others primarily as sexual objects, increasing the likelihood of using coercive tactics to obtain sex or reduce sensitivity to verbal and nonverbal reluctance. While few studies have tested these predictions, some evidence exists. For instance, Wright et al. (2021) found that among heterosexual men in the United States, those with more impersonal attitudes towards sex and greater exposure to pornography depicting dominance and aggression were more likely to engage in sexually aggressive behavior.

The relationship between sexual permissiveness and sexual behavior has also been explored in the context of risky behaviors. Wright and Vangeel (2019), using data from the United States General Social Survey gathered between 1990 and 2016, found that individuals with more permissive sexual attitudes reported a greater number of sexual partners within a year. In contrast, a study of French college students by Gana and Arshakyan (2023) revealed that more permissive sexual attitudes predicted increased condom use, suggesting that sociosexual attitudes do not necessarily translate into risky sexual behavior.

### 1.4. Pornography Consumption, Sexual Double Standard, and Sexual Behavior

The sexual double standard refers to differing evaluations of comparable sexual behaviors based on gender, typically granting men greater sexual freedom than women, particularly concerning casual or premarital sex and the number of sexual partners (Milhausen & Herold, 2002). More recent conceptualizations extend this definition to include behaviors that are not only permitted, but also expected, and valued differently for men and women (Endendijk et al., 2020). The sexual double standard has been a focal point of research. Early studies measured permissiveness towards premarital sex using a scale with two dimensions: permissiveness, addressing the role of affection in sexual activities, and equalitarianism, examining how these activities were evaluated differently for men and women (Reiss, 1964).

Traditionally, heterosexual men have been expected to be dominant and initiate sexual activities, while women have been expected to be submissive and passive (Endendijk et al., 2022). This heterosexual script is reinforced through media portrayals (Aubrey et al., 2020; Ward & Grower, 2020). As Zaikman and Marks (2017) noted, media often depict female sexuality as “dirty, dangerous, or taboo”, while framing male sexuality as “normal, proper, or good” (p. 416). Mainstream pornography perpetuates these dynamics, frequently depicting women in submissive roles and as primary targets of violence (Richardson, 2018; Fritz & Paul, 2017). A study among adolescents and emerging adults found that exposure to sexual media and sexualized music videos was associated with a stronger endorsement of traditional sexual double standard norms among boys, but not girls (Endendijk et al., 2022). However, the effect of pornography consumption was not significant, potentially due to a lack of identification with porn actors compared to social media figures. In contrast, Ortiz et al. (2016) reported that pornography consumption among college students was negatively associated with the endorsement of the sexual double standard. This relationship, however, was partially mediated by the perceived acceptability and impact of pornography. Specifically, individuals who consumed pornography more frequently tended to perceive it as a more acceptable and positive activity, which was associated with lower agreement with the sexual double standard. Overall, these findings suggest an influential role of media representation in societal perceptions of gender roles and sexuality, while highlighting the importance of personal attitudes.

Prior research has also examined the connection between adherence to the sexual double standard and sexual behavior. For example, Lefkowitz et al. (2014) found that, among college students, men who adhered to the sexual double standard reported a greater number of lifetime sexual partners and held fewer negative attitudes towards condom use. In contrast, women who adhered more strongly to the sexual double standard were more likely to exhibit negative attitudes towards condom use. However, for women, adherence to the sexual double standard was not associated with the number of lifetime sexual partners. In a systematic review, Álvarez-Muelas et al. (2020a) reported that adherence to the sexual double standard can be particularly harmful for women, as it may compromise their agency. In line with this, Jozkowski et al. (2017) found that women endorsing these attitudes feared slut-shaming, and were more likely to consent to undesired sexual encounters to protect men’s egos. Similarly, Farvid et al. (2017) indicated that women, particularly those who were influenced by the sexual double standard, avoided disclosing details about their sex lives to protect their reputations.

Despite these insights, few studies have explored the relationship between the sexual double standard and violent sexual behavior. Eaton and Matamala (2014) found that, among college students, stronger adherence to the sexual double standard was associated with men’s perpetration of verbal sexual coercion, but not women’s. More recently, Chadwick-Brown and Endendijk (2024) used a sample of adolescents and emerging adults to show that stronger adherence to the sexual double standard increased men’s odds of perpetrating sexual coercion while reducing those odds for women.

## 2. Method

### 2.1. Participants and Procedure

Data were collected online between 19 January and 14 February 2024, using the Verian opt-in panel (formerly Kantar) in Spain, a platform that allows individuals to voluntarily enroll in surveys and research studies. Quotas for gender and age were used to obtain a sample distribution similar to the population of Castilla-La Mancha (see Appendix A for a comparison between the achieved sample and population data). Of the panelists invited to participate, 36.1% completed the questionnaire and received points redeemable for gifts as a token of appreciation for their time. The final sample consisted of 1003 panelists residing in Castilla-La Mancha (50.7% men), with ages ranging from 16 to 84 (*M* = 45.5, *SD* = 14.3).

Before administering the survey, a sequential pretest was conducted between December 2023 and January 2024. This pretest included expert reviews (*n* = 4) and online cognitive interviews (one round, *n* = 5) (additional details are provided in Appendix B). The survey, administered in Spanish, included up to 57 items^1^ measuring public attitudes towards sexuality and the traditional sexual double standard, as well as questions on sexual permissiveness, pornography consumption, and engagement in violent sexual practices, among other topics. On average, the survey took 12 min to complete (*M* = 12.2; *mdn* = 10.9; *SD* = 5.8). The study procedures were approved by the Ethics Committee of the University of Castilla-La Mancha (protocol code: CEIS-2024-23586).

### 2.2. Measures

#### 2.2.1. Outcome Variables

***Perpetrating Violent Sexual Practices.*** These items were administered only to respondents who had engaged in sexual relationships.^2^ Participants were asked whether they had ever perpetrated four specific violent behaviors during sexual encounters: (1) spanking the buttocks, (2) pulling hair, (3) using obscene language, and (4) forcefully grabbing the neck. Responses were recorded on a dichotomous yes/no scale, with an additional “I prefer not to answer” option. The responses were summed to create an overall score for perpetrating violent sexual practices (range 0–4), with higher scores indicating the perpetration of more violent sexual practices.

***Experiencing Violent Sexual Practices.*** These items, also limited to respondents who had engaged in sexual relationships,^3^ were presented immediately after the questions about perpetration. Respondents were asked whether they had ever experienced the same four violent behaviors during sexual encounters: (1) spanking the buttocks, (2) pulling hair, (3) using obscene language, and (4) forcefully grabbing the neck. Responses were recorded on a dichotomous yes/no scale, with an additional “I prefer not to answer” option. The responses were summed to create an overall score for experiencing violent sexual practices (range 0–4), with higher scores indicating the experience of a wider range of violent sexual practices.

#### 2.2.2. Mediator Variables

***Sexual Permissiveness*** (*α* = .79). This construct was measured using a subscale of the Brief Sexual Attitudes Scale (Hendrick et al., 2006), which consists of five items assessing liberal versus conservative attitudes towards sexual activities. Statements include “Casual sex is acceptable” and “One-night stands are sometimes enjoyable”. Each item was rated on a 5-point agree/disagree scale. Responses were averaged to create an overall score (range 1–5), with higher scores indicating greater sexual permissiveness.

***Acceptance of Sexual Double Standard*** (*α* = .84). This construct was measured using a subscale of the Sexual Double Standard Scale (SDSS, Muehlenhard & Quackenbush, 2011), as adapted to Spanish by Gómez-Berrocal et al. (2019). The subscale consisted of five items reflecting adherence to the traditional sexual double standard. Sample statements include “Men should be more sexually experienced than women” and “Women who have sex on the first date are ‘easy’”. Respondents rated each item on a 5-point agree/disagree scale, and responses were averaged to create an overall score (range 1–5), with higher scores indicating greater acceptance of the sexual double standard.

#### 2.2.3. Explanatory and Control Variables

***Pornography Consumption.*** Respondents were asked: “Throughout your life, have you ever consumed pornography?”. Given the broad nature of the term “pornography”, we provided the following definition to respondents before administering the question: “By pornography, we refer to all materials (i.e., texts, audios, images, and videos) that depict sexual or erotic acts used to provoke sexual arousal in the user”. If they answered “yes”, they were subsequently asked about the frequency of their consumption over the past 12 months. The first question used a dichotomous yes/no scale, with an additional “I prefer not to answer” option. The second question used an 8-point scale ranging from *never* to *every day*, with the “I prefer not to answer” option also available.

***Sociodemographic Variables*** included gender, age (in years), education (categorized as school graduate or less; high school or technical school; college graduate; master or doctorate), political orientation (measured on a scale ranging from 0—left to 10—right), and sexual orientation (heterosexual; non-heterosexual).

### 2.3. Analytic Strategy

**Figure 1:**
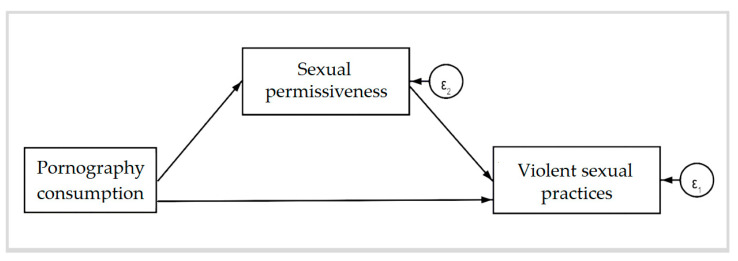
A path diagram showing sexual permissiveness as a mediator between pornography consumption and violent sexual practices.

Statistical analyses were conducted using Stata 16. Descriptive statistics were first computed for all study variables. To explore the relationships between perpetrating and experiencing violent sexual practices, pornography consumption, sexual permissiveness, and acceptance of the sexual double standard, Pearson bivariate correlations were performed. The analyses revealed no significant association between pornography consumption and acceptance of the sexual double standard. Based on this finding, we tested the hypothesis that sexual permissiveness would mediate the relationship between pornography consumption and both perpetrating and experiencing violent sexual practices during sexual relationships, as illustrated in Figure 1.

To test this hypothesis, a mediation analysis was conducted using the “medeff” command in Stata (Hicks & Tingley, 2011, 2012), which estimates both direct and indirect effects of pornography consumption on violent sexual behaviors. Two linear regression models were estimated, controlling for gender, age, education, political orientation, and sexual orientation. In the first model, sexual permissiveness (the mediator) was regressed on pornography consumption. In the second step, separate models were estimated for perpetration and victimization, in which engagement in violent sexual practices was regressed on both pornography consumption and sexual permissiveness. Variance Inflation Factors (VIF) were within acceptable limits, indicating no multicollinearity issues (1.01 ≤ *VIF* ≤ 1.40).

## 3. Results

### 3.1. Summary of Study Variables

Table 1 provides summary statistics for the study variables. Slightly over two in five respondents (41.8%) had completed a college education. On average, the sample leaned towards the political center (*M* = 5.31 on a scale from 0—left to 10—right), and the vast majority of respondents (86.6%) identified as heterosexual.

Lifetime pornography consumption was high, with 72.5% of respondents reporting that they had consumed pornography at some point in their lives. However, nearly half of the sample (49.6%) reported no pornography consumption in the last 12 months. Regarding sexually violent practices, respondents reported perpetrating an average of one sexually violent practice during sexual encounters (*M* = 0.80, range 0–4), a figure similar to the average number of sexually violent practices experienced (*M* = 0.97, range 0–4). The findings also indicate a moderate level of sexual permissiveness among the sample (*M* = 3.05 on a scale from 1 to 5) and a low acceptance of the traditional sexual double standard (*M* = 1.70 on a scale from 1 to 5).

### 3.2. Engagement in Sexually Violent Practices During Sexual Encounters

Table 2 presents the prevalence of respondents who reported perpetrating sexually violent practices during sexual encounters. Approximately half of the sample (53.6%) indicated that they had not engaged in any such practices. This was followed by 24.4% who reported having perpetrated one sexually violent practice, 12.7% who reported two practices, and 9.3% who reported three or more practices. Among respondents who reported two or more sexually violent practices, the most common combination was spanking the buttocks, pulling hair, and using obscene language (22.0%). This was followed by spanking the buttocks and using obscene language (21.2%) and spanking the buttocks and pulling hair (20.3%).

The results revealed gender differences (*X*^2^ = 25.41, *p* < .001), with a small effect size (*V* = .16). While approximately six in ten women (59.3%) reported not having perpetrated any of the practices, this figure was lower for men (48.0%). Gender differences were most pronounced in the categories of three and four sexually violent behaviors, where the percentages differed by approximately four percentage points.

Table 2 also summarizes the prevalence of respondents who reported experiencing sexually violent practices. Approximately half of the sample (49.7%) indicated that they had not experienced any such practices during sexual encounters. This was followed by 22.1% who reported experiencing one practice, 15.2% who reported two practices, and 13.0% who reported three or more practices. The most common combination of sexually violent practices was spanking the buttocks and using obscene language (26.8%). Other frequent combinations included spanking the buttocks, pulling hair, using obscene language, and forcefully grabbing the neck (17.1%), and spanking the buttocks, pulling hair, and using obscene language (16.4%).

Significant gender differences were also observed in experiences of sexually violent practices (*X*^2^ = 30.58, *p* < .001), with a modest effect size (*V* = .18). While 54.5% of men reported no experience with any of the practices, only 44.7% of women reported the same. Additionally, 24.3% of men reported experiencing one sexually violent behavior, compared to 20.0% of women. In all other categories, women consistently reported higher frequencies of experiencing sexually violent practices than men (see Table 2).

### 3.3. Acceptance of Sexual Double Standard and Sexual Permissiveness

The overall acceptance of the sexual double standard was low, with an average score of 1.70 (*SD* = 0.76) on a scale ranging from 1 (strongly disagree) to 5 (strongly agree). Men were more likely than women to accept the sexual double standard (t = 6.61, df = 1001, *p* < .001), with a medium-sized effect (*d* = 0.42). Women scored an average of 1.55 (*SD* = 0.68) on this scale, compared to men, who scored higher, at 1.86 (*SD* = 0.80).

Among the full sample, the items receiving the highest levels of agreement were “Women who have sex on the first date are ‘easy’” (58.9%) and “I question the character of women who have a lot of sexual partners” (51.3%). Conversely, the items with the lowest levels of agreement were “Men should be more sexually experienced than women” (21.1%) and “Women who initiate sex are too aggressive” (32.0%).

A comparison of the item-level responses revealed differences between women and men on most items, with men agreeing more than women, except for the items “It’s worse for a woman to sleep around than it is for a man” and “Women who initiate sex are too aggressive” (see Table 3). Effect sizes were small, with the largest differences observed for the items “Women who have sex on the first date are ‘easy’” (*V* = .22) and “Men should be more sexually experienced than women” (*V* = .21).

Regarding sexual permissiveness, the overall score was moderate (*M* = 3.05 on a scale from 1 “strongly disagree” to 5 “strongly agree”). Men reported higher levels of sexual permissiveness than women (t = 7.83, df = 1001, *p* < .001), with a medium-sized effect (*d* = 0.50). Women scored an average of 2.83 (*SD* = 0.84), while men scored higher, at 3.27 (*SD* = 0.95).

Across the full sample, the items with the highest levels of agreement were “Casual sex is acceptable” (57.8%) and “Sex as a simple exchange of favors is okay if both people agree to it” (53.0%). In contrast, the item with the lowest level of agreement was “I would like to have sex with many partners” (14.2%). Differences between women and men were observed across all items, except for “Casual sex is acceptable”. For most items, men agreed more than women; however, women agreed more than men on “Sex as a simple exchange of favors is okay if both people agree to it” (58.9% versus 49.1%). The largest differences were observed for the items “I would like to have sex with many partners” (*V* = .37) and “I do need to be committed to a person to have sex with them” (*V* = .23).

### 3.4. Pornography Consumption

As previously noted, 72.5% of respondents reported having consumed pornography at some point in their lives. However, gender differences were observed (*X*^2^ = 113.11, *p* < .001), with a medium-sized effect (*V* = −.34). A higher proportion of men (87.5%) reported lifetime pornography consumption compared to women (56.8%).

Nearly half of the sample (49.6%) indicated that they had not consumed pornography in the last 12 months. This was followed by those who had consumed pornography several times during the last 12 months (15.2%) and several times a month (12.0%). Gender differences in pornography consumption over the last 12 months were also significant (*X*^2^ = 213.52, *p* < .001), with a medium-sized effect (*V* = .47). While 70.1% of women reported not consuming pornography in the last 12 months, this percentage was substantially lower for men, at 29.3%. Differences were particularly evident in the categories of several times a month and several times a week. Specifically, 20.0% of men reported consuming pornography several times a month, compared to only 4.0% of women. In the category of several times a week, 14.6% of men reported this frequency, compared to just 1.0% of women.

### 3.5. Relationships Among Study Variables

The correlational analysis results, as shown in Table 4, reveal a strong positive association between perpetrating and experiencing sexually violent practices (*rxy* = .658, *p* < .001). Perpetration of violent sexual practices was unrelated to acceptance of the sexual double standard (*rxy* = −.029, *p* = .371). However, a moderate positive correlation was found between perpetration and sexual permissiveness (*rxy* = .295, *p* < .001). Additionally, perpetrating violent sexual practices was positively correlated with both lifetime pornography consumption and the frequency of pornography consumption in the last 12 months (see Table 4). Similarly, experiencing violent sexual practices was negatively associated with acceptance of the sexual double standard (*rxy* = −.113, *p* < .001), and positively associated with sexual permissiveness (*rxy* = .221, *p* < .001). Similarly to perpetration, experiencing violent sexual practices was positively associated with both lifetime pornography consumption and the frequency of pornography consumption in the last 12 months (see Table 4).

### 3.6. Role of Sexual Permissiveness in Relationship Between Pornography Consumption and Violent Sexual Practices

To investigate the relationship between pornography consumption and engagement in violent sexual practices, linear regression models were estimated, controlling for relevant sociodemographic variables (see Section 2 for details). These models consistently indicated that pornography consumption, both lifetime and in the past 12 months, is associated with engagement in violent sexual practices during sexual encounters.

As shown in Table 5, lifetime pornography consumption was associated with both perpetrating (*b* = 0.60, *p* < .001) and experiencing (*b* = 0.13, *p* < .001) violent sexual practices. Both lifetime pornography consumption and the frequency of consumption in the last 12 months predicted the perpetration of violent sexual practices (see Table 6). Sexual permissiveness mediated this relationship, explaining approximately 20% of the association between pornography consumption and the perpetration of sexually violent practices. Gender-disaggregated analysis, detailed in Appendix C, revealed a stronger mediation effect for males. Specifically, the mediation effect for lifetime pornography consumption was 29.4% for men and 13.5% for women, while for the frequency of consumption in the past 12 months, the mediation effect was 28.7% for men and 9.7% for women.

Pornography consumption, both lifetime and in the past 12 months, was also associated with experiencing sexually violent practices during sexual encounters (see Table 5). Similarly to perpetration, sexual permissiveness mediated the relationship between pornography consumption and experiencing violent sexual practices (see Table 6). Gender-disaggregated analysis, available in Appendix C, indicated a stronger mediation effect for men. Specifically, the mediation effect for lifetime pornography consumption was 36.9% for men and 17.0% for women. For the frequency of pornography consumption, the mediation effect was 30.9% for men and 12.5% for women.

## 4. Discussion

This study explores the relationship between pornography consumption and violent sexual practices in a central region of Spain, focusing on the potential mediating roles of sexual permissiveness and the sexual double standard. As expected, pornography consumption was associated with both the perpetration and experience of violent sexual practices (RQ_1_). However, only sexual permissiveness emerged as a significant mediator, while the sexual double standard showed no significant association with pornography consumption (RQ_2_).

### 4.1. Pornography Consumption and Violent Sexual Behavior

Overall, rates of lifetime pornography consumption were high among respondents. This aligns with previous national and international studies indicating that 8 in 10 individuals have consumed pornography at some point (Ballester-Arnal et al., 2023; Solano et al., 2018). As in prior research, men reported higher levels of pornography consumption than women, particularly when considering recent consumption (Gomez-Miguel et al., 2023; Sanz-Barbero et al., 2024).

More than half of the respondents reported never having perpetrated any of the examined violent sexual practices, and nearly half indicated that they had not experienced such behaviors in their sexual relationships. However, given the sensitivity of the topic, social desirability bias may have influenced these self-reported data, potentially leading to under-reporting of both perpetration and victimization. Nonetheless, the prevalence rates found in this study are similar to those reported by Herbenick et al. (2021), who found that participation in activities like spanking, choking, and name-calling ranged from 60.2% to 15.7% in a cross-sectional survey of undergraduate college students in the United States.

Gender differences emerged in both the perpetration and experience of violent sexual practices. Men were more likely to report perpetrating such behaviors, while women more often reported being on the receiving end. This pattern is consistent with findings by Herbenick et al. (2023), which showed that men more often reported choking a partner, whereas women more often reported being choked. These dynamics suggest a dominant sexual script characterized by the subordination and domination of women. Previous studies have found that women are typically targets of sexually violent practices in pornographic scenes, with spanking and hair-pulling being among the most common forms of physical aggression (Fritz & Paul, 2017; Fritz et al., 2020; Klaassen & Peter, 2015).

Individuals who reported a higher prevalence of perpetrating violent sexual acts were also more likely to have experienced them on the receiving end. This suggests that the normalization of violent behavior in sexual relationships is not limited to a specific role; rather, it appears to foster a cyclical pattern of violence. Those who experience such behaviors may become perpetrators in future encounters, reinforcing the normalization of violent practices in sexual relationships. Furthermore, as expected, both perpetrating and experiencing violent sexual practices were associated with pornography consumption, whether such consumption occurred over a lifetime or in the past 12 months. As mentioned, pornography eroticizes violent sexual practices, with such depictions being common in mainstream content and frequently portrayed as pleasurable. These factors are central to the 3AM Theory (Wright, 2011), which posits that the frequent portrayal of violent behaviors and the associated reward system contribute to the Acquisition and Application of these sexual scripts, respectively. Additionally, according to this theory, repeated exposure to sexualized materials fosters the internalization of these scripts, while recent exposure increases the likelihood of Activating them. These findings suggest that the sexual script embedded in pornography, which systematically depicts women as targets of male aggression, might influence real-world sexual behavior (Fritz & Paul, 2017).

### 4.2. Sexual Double Standard, Sexual Behavior, and Pornography Consumption

Respondents in this study exhibited relatively low acceptance of the sexual double standard, reflecting broader social trends in Western countries like Spain, where egalitarian values are increasingly prevalent. These findings are aligned with those of Gómez-Berrocal et al. (2019), who observed similar patterns in a Spanish heterosexual sample. Additionally, Álvarez-Muelas et al. (2020b) reported that individuals in Spain generally apply the same evaluative criteria to men’s and women’s sexual behavior, a trend further supported by recent national surveys (Centro de Investigaciones Sociológicas, 2024). Low acceptance of the sexual double standard suggests a societal context that is supportive of female sexual agency, potentially reducing slut-shaming and improving women’s sexual experiences in terms of both pleasure and safety. For instance, research has shown that women who adhere more strongly to sexual double standard norms are less likely to use condoms as sexual protection (Álvarez-Muelas et al., 2020a). Moreover, reduced acceptance of the sexual double standard may challenge expectations of male sexual dominance and initiation, while recognizing male sexual vulnerabilities (Endendijk et al., 2020). In this sense, low acceptance of the sexual double standard can serve as an indicator of an egalitarian society, reflecting progress in dismantling various forms of sexism.

Despite evidence suggesting a null effect of gender on acceptance of sexual double standard norms (Endendijk et al., 2020), our findings reveal that men exhibited higher levels of acceptance than women. This is consistent with Álvarez-Muelas et al. (2020b), who found that men tend to support other men in sustaining gender privileges in Spanish society. In response to feminist advances, a backlash may be taking shape, reflecting concerns over the perceived erosion of traditional masculinity (Ranea, 2021). Men adhering to traditional values may be more receptive to narratives promoting these ideals (e.g., body count, tradwife movements, men as breadwinners) (Mattheis, 2021). Consequently, men who endorse the sexual double standard may be more inclined to support narratives that subordinate women, undermine their sexual agency, and legitimize violence against them.

Regarding engagement in violent sexual practices, we found that acceptance of the sexual double standard was not associated with perpetrating such acts. However, it was weakly related to experiencing them. In contrast, acceptance of the sexual double standard was not linked to pornography consumption, whether measured over a lifetime or in the past 12 months. This finding aligns with previous research. For instance, Endendijk et al. (2022) found no relationship between pornography use and adherence to sexual double standard norms among Dutch adolescents and emerging adults. They suggested that other forms of media, such as social media, may have a stronger influence on these norms, as audiences might find them more relatable and easily applicable to their own lives compared to pornographic content. Another possible explanation is that, despite the frequent subordination and degradation of women in pornography, such content often includes portrayals of both men and women engaging in behaviors that are traditionally considered to be masculine. These portrayals may conflict with the notion that men should always initiate sexual encounters or be permitted multiple partners, while women are discouraged from exhibiting such behaviors. Contrary to the restricted sexual agency prescribed by the sexual double standard, pornography often features content that challenges these norms (e.g., women having sex on the first date, non-monogamous women, sexually experienced women). This contradiction may help to explain the lack of a significant relationship between adherence to the sexual double standard and pornography consumption.

### 4.3. Sexual Permissiveness, Sexual Behavior, and Pornography Consumption

Respondents reported moderate levels of sexual permissiveness, with men exhibiting more permissive attitudes than women, except regarding the acceptability of casual sex, which received the highest support without significant gender differences. These findings reflect broader trends in Spain, where over half of both men and women endorse the acceptability of sex without love or engaging in multiple simultaneous sexual and/or affective relationships (Centro de Investigaciones Sociológicas, 2023). The results suggest that women generally hold less permissive attitudes towards sexual matters compared to men, likely due to differing gender socialization processes. Wright and Vangeel (2019), analyzing data from the United States General Social Survey collected between 1990 and 2016, found that the relationship between pornography consumption and sexual permissiveness was stronger for men than for women. They discussed that women appear less interested in casual sex, potentially due to the heightened risk of disrespect or stigmatization of sexual permissiveness (England & Bearak, 2014). Women face greater social penalties for having an active sex life or engaging in “male-typical” sexual behaviors. As Farvid et al. (2017) noted, even women who are critical of these social constraints often avoid disclosing casual sex experiences, in order to maintain a respectable sexual image and differentiate themselves from women with stigmatized sexual reputations. Blake et al. (2016) further highlighted that both men and women who perceive women as more sexually open are more likely to question their agency and view them as more vulnerable to sexual victimization. Consequently, women may adopt a more conservative sexual approach as a protective strategy against potential victimization and reputational harm.

When examining violent sexual practices, sexual permissiveness was linked to both perpetrating and experiencing such behaviors. Respondents with higher levels of sexual permissiveness reported engaging in and experiencing a broader range of violent sexual practices within relationships. This may reflect fewer rigid boundaries regarding sexually acceptable behavior among permissive individuals. These practices are frequently portrayed in popular culture not only as pleasurable, but also as markers of sexual openness (Kummerow, 2016). As a result, such behaviors may become normalized and accepted as conventional sexual practices, despite their violent nature.

Sexual permissiveness was also associated with pornography consumption, both over a lifetime and in the short term. Respondents with higher levels of permissiveness were more likely to have consumed pornography at some point, and reported increased consumption in the past 12 months. These findings align with previous research (Tokunaga et al., 2019; Wright & Vangeel, 2019), and were anticipated, given that pornography often eroticizes casual, impersonal, and detached sexual encounters (Miller & McBain, 2021; Rasmussen et al., 2019; Tokunaga et al., 2019). It has been suggested that the effects of pornography consumption on sexually permissive attitudes may persist over time (Wright, 2013a), which could explain the association with both lifetime and recent consumption. However, given the design of our study, causality cannot be established. It is plausible that sexually permissive individuals are more comfortable with using pornography, supporting the Selective Exposure Theory. However, attributing these findings solely to selective exposure contradicts the broader body of evidence. Multiple studies have demonstrated both unidirectional and reciprocal effects of pornography on attitudes, beliefs, and behavior (Krahé et al., 2024; Malamuth et al., 2000, 2012), while few have supported selective exposure as the sole explanation (Wright et al., 2021).

### 4.4. Mediation Effect

Our findings indicate that sexually permissive attitudes, rather than the sexual double standard, mediate the relationship between pornography consumption and engagement in violent sexual practices. Specifically, sexual permissiveness mediated the relationship between both lifetime and recent pornography consumption and the perpetration of violent sexual practices. Similarly, it mediated the association between pornography consumption—both lifetime and recent—and the number of violent sexual practices experienced. According to the 3AM Theory (Wright, 2011), pre-existing individual attitudes, such as sexual permissiveness, facilitate the activation of acquired scripts by making them more accessible in an individual’s memory. As noted, these violent practices are often associated with sexual openness. The compatibility between pornographic sexual scripts and existing attitudes further promotes their activation. These results suggest that sexual permissiveness may act as a catalyst for engaging in violent sexual behaviors. Individuals with more permissive attitudes may adopt a more lenient perspective on what is considered enjoyable, normal, and justifiable within sexual relationships, thereby encouraging the enactment of violent practices. In contrast, the non-significant relationship between pornography consumption and the sexual double standard may reflect the portrayal of men and women engaging in similar sexual behaviors regardless of gender, as discussed previously.

A gender-disaggregated analysis revealed that the mediation effect of sexual permissiveness was stronger for men than for women. This difference may be attributed to men’s higher rates of pornography consumption, both lifetime and in the past 12 months. Frequent exposure to pornography may contribute to the internalization of a violent sexual script, a process reinforced by permissive sexual attitudes prevalent in societies such as Spain. Ultimately, this promotes the normalization and acceptance of such behaviors, leading men to engage in them during sexual encounters to a greater extent than women, who reported lower rates of lifetime and recent pornography consumption.

Overall, our results support the 3AM Theory (Wright, 2011), which posits that media consumption, particularly pornography, influences sexual behavior. However, this relationship is mediated by specific sexual norms, which, together, contribute to engagement in violent sexual practices. This highlights the complex interplay between media exposure and sexual behavior, underscoring the role of underlying sexual attitudes and norms. Importantly, our findings emphasize the need to address sexual permissiveness in interventions aimed at reducing violent sexual behaviors and promoting healthier sexual dynamics.

### 4.5. Limitations

Despite its valuable contributions, this study has several limitations. First, the sample was obtained from a non-probabilistic panel, limiting the generalizability of the findings. Additionally, the data were collected from a specific region in Spain (Castilla-La Mancha), and may not fully represent the broader Spanish population. Notably, the sample deviated from the populations of both Castilla-La Mancha and Spain, with an over-representation of individuals with higher education levels, a common issue in both probability- and non-probability-based samples (additional details are available in Appendix A). Furthermore, the requirement for parental consent for 16-year-old respondents, who are below the legal age of majority, may have reduced participation in this age group.

Second, the study did not analyze the frequency, timing, or dynamics of violent sexual practices, preventing an understanding of how often these practices occur or the context in which they take place. It also did not address aspects related to consent or the enjoyment derived from violent practices. Future research should explore these factors to better understand the pathways linking pornography consumption to engagement in violent sexual behaviors. Third, the study did not differentiate between specific types of pornography. Future research should include a range of pornographic categories to better understand how different types of content influence sexual behavior. Fourth, the study did not collect data on the gender of respondents’ sexual partners in relation to the violent sexual practices they reported, limiting our ability to explore experiences across different sexual orientations. Future studies with larger sample sizes should investigate differences in violent sexual practices among LGBTQ+ individuals.

Lastly, due to the correlational nature of the study, we cannot establish whether a third variable may influence the relationship between pornography consumption and engagement in violent sexual practices, or whether reverse causality may be at play. As such, this limitation should be considered when interpreting the findings, and caution is advised when drawing conclusions about causal relationships. Future research should implement longitudinal designs to gain a clearer understanding of the causal relationships between pornography consumption and violent sexual practices. Additionally, examining potential mediating and moderating variables, such as individual psychological traits or relationship dynamics, could offer valuable insights into the mechanisms underlying these associations.

## 5. Conclusions

Our study highlights the widespread prevalence of pornography consumption in Spain, particularly among men, with a significant gender gap in both lifetime and recent consumption. Men reported substantially higher levels of pornography use compared to women. While most respondents did not report engaging in violent sexual practices, pornography consumers were more likely to report both perpetrating and experiencing such behaviors, including spanking, hair-pulling, using obscene language, and forcefully grabbing the neck. These findings can be attributed to several factors, including the normalization and erotization of aggressive sexual behaviors in pornography (Wright, 2011). Mainstream pornography often portrays these practices as arousing and consensual, leading individuals to internalize them as acceptable. This blurring of fantasy and reality increases the likelihood of incorporating these behaviors into sexual encounters as part of perceived normal sexual expression. Our findings also highlight notable gender differences: men were more likely to perpetrate violent practices, while women were more likely to experience them. This dynamic is consistent with traditional power imbalances often portrayed in pornography, where men are depicted as dominant and women as submissive. Such depictions can reinforce harmful stereotypes about gender roles in sexual relationships, perpetuating violence and inequality in real-word sexual behavior.

Additionally, the analysis emphasizes the critical role of sexual permissiveness as a mediator between pornography consumption and engagement in violent sexual practices, particularly among men. Individuals with more permissive sexual attitudes may be more likely to view violent sexual behaviors as acceptable, increasing their tendency to both engage in and experience these practices. This effect was particularly pronounced in men, suggesting that gender-specific attitudes towards sexual permissiveness and aggression play a central role in shaping these behaviors. In contrast, our study found no relationship between the sexual double standard and pornography consumption. This suggests that the sexual double standard may not play a central role in influencing the link between pornography consumption and violent sexual practices. Instead, sexual permissiveness, as a broader and more encompassing value, appears to be a more influential mediator in this relationship.

These findings call for action to address the impact of pornography consumption on sexual behavior and attitudes. As pornography continues to shape sexual norms, educational and intervention programs must focus on confronting its role in normalizing violence and promoting harmful sexual practices. While these findings are particularly relevant to preventing sexual violence, they also highlight the importance of addressing intimate partner and domestic violence, as both are closely linked to the normalization of violent and aggressive behavior in intimate relationships. The harmful dynamics promoted by pornography—such as power imbalances, the subordination of women, and the eroticization of aggression—often extend into romantic relationships, reinforcing cycles of violence. Tackling these issues within the context of intimate partner and domestic violence can foster a more comprehensive and critical understanding of these behaviors, ultimately supporting efforts to prevent all forms of sexual violence.

Policies aimed at preventing early access to pornographic materials, along with educational and intervention programs, could be an effective strategy for promoting healthier, more egalitarian, and consensual sexual practices. It is also important to address other contributing factors, such as attitudes, which have been shown to mediate the relationship between pornography consumption and engagement in violent sexual practices. The complexity of these results underscores the need for multifaceted solutions in the prevention of sexual violence. We believe that our study emphasizes the visibility of inherently violent behaviors that are highly normalized in society. Individuals may identify more closely with these practices than with scenarios that are typically examined in other studies, such as sexual aggression. This distinction represents a key strength of our study, as it encourages a critical social analysis of these behaviors, making them more relatable and potentially influencing real-world experiences.

## Figures and Tables

**Table 1:** Summary statistics for the study variables.

Variable	% (*n*)	*M* (*SD*)
**Lifetime perpetration of sexually violent practices** (range 0 *none*–4 *four practices*)		0.80 (1.05)
**Lifetime experience of sexually violent practices** (range 0 *none*–4 *four practices*)		0.97 (1.19)
**Sexual permissiveness** (*α* = .79) (range 1–5)		3.05 (0.93)
**Acceptance of sexual double standard** (*α* = .84) (range 1–5)		1.70 (0.76)
**Lifetime pornography consumption**	72.5% (692)	
**Frequency of pornography consumption in last 12 months**		
Never	49.6% (479)	
Once in last 12 months	5.3% (51)	
Several times in last 12 months	15.2% (147)	
Once a month	5.2% (50)	
Several times a month	12.0% (116)	
Once a week	3.9% (38)	
Several times a week	7.9% (76)	
Every day	0.9% (9)	
**Gender**		
Men	50.7% (509)	
Women	49.3% (494)	
**Age** (in years)		45.51 (14.31)
16–24 years old	10.4% (104)	
25–44 years old	37.5% (376)	
45–65 years old	43.0% (431)	
66+ years old	9.2% (92)	
**Education**		
School graduate or less	16.6% (166)	
High school or technical school	41.6% (418)	
College graduate	32.0% (321)	
Master or doctorate	9.8% (98)	
**Political orientation** (0 *left*–10 *right*)		5.31 (2.44)
**Sexual orientation**		
Heterosexual	86.6% (850)	
Non-heterosexual	12.4% (131)	

Note: The analytical sample for the variables “sexual orientation”, “lifetime perpetration of sexually violent practices”, “lifetime experience of sexually violent practices”, “lifetime pornography consumption”, and “frequency of pornography consumption in the last 12 months” was reduced, due to some respondents selecting “Prefer not to answer”. *M* = mean, *SD* = standard deviation.

**Table 2:** Engagement in sexually violent practices by gender.

Count	Full Sample (*N* = 1003) % (*n*)	Men (*N* = 509) % (*n*)	Women (*N* = 404) % (*n*)	*X* ^2^	Cramer’s *V*
***Perpetration of sexually violent practices***					
0	53.6% (527)	48.0% (240)	59.3% (287)	25.41 ***	.16
1	24.4% (240)	25.2% (126)	23.6% (114)		
2	12.7% (125)	13.8% (69)	11.6% (56)		
3	7.3% (72)	9.4% (47)	5.2% (25)		
4	2.0% (20)	3.6% (18)	0.4% (2)		
***Experience of sexually violent practices***					
0	49.7% (487)	54.5% (272)	44.7% (215)	30.58 ***	.18
1	22.1% (217)	24.3% (121)	20.0% (96)		
2	15.2% (149)	13.4% (67)	17.1% (82)		
3	7.8% (76)	5.2% (26)	10.4% (50)		
4	5.2% (51)	2.6% (13)	7.9% (38)		

Note: The total frequencies do not sum to 1003, due non-substantive responses. *** *p* ≤ .001.

**Table 3:** Descriptive statistics for the Sexual Double Standard Scale and sexual permissiveness.

Item	Full Sample (*N* = 1003) % (*n*)	Men (*N* = 509) % (*n*)	Women (*N* = 404) % (*n*)	*X* ^2^	Cramer’s *V*
***Sexual Double Standard Scale***					
It’s worse for a woman to sleep around than it is for a man.	36.6% (41)	28.7% (23)	45.3% (18)	27.94 ***	.17
Women who initiate sex are too aggressive.	32.0% (47)	31.9% (30)	32.8% (17)	25.35 ***	.16
I question the character of women who have a lot of sexual partners.	51.3% (76)	59.4% (54)	40.6% (22)	34.00 ***	.18
Men should be more sexually experienced than women.	21.1% (29)	28.7% (23)	10.9% (6)	43.12 ***	.21
Women who have sex on the first date are ‘easy’.	58.9% (76)	51.3% (43)	70.3% (33)	49.50 ***	.22
***Sexual Permissiveness Scale***					
I do not need to be committed to a person to have sex with them.	36.7% (370)	38.5% (230)	34.2% (140)	51.74 ***	.23
Casual sex is acceptable.	57.8% (577)	52.4% (309)	65.6% (268)	5.33	.07
I would like to have sex with many partners.	14.2% (148)	20.5% (126)	5.02% (22)	137.30 ***	.37
One-night stands are sometimes enjoyable.	38.2% (395)	39.6% (241)	36.3% (154)	28.47 ***	.17
Sex as a simple exchange of favors is okay if both people agree to it.	53.0% (531)	49.1% (291)	58.9% (240)	10.84 *	.10

Note: The items that composed the Sexual Double Standard Scale are from the Sexual Double Standard Scale (Gómez-Berrocal et al., 2019). The items that composed the Sexual Permissiveness Scale are from the Brief Sexual Attitudes Scale (Hendrick et al., 2006). The percentages of agreement have been calculated by summing the response categories “agree” and “totally agree”. *** *p* ≤ .001, * *p* ≤ .05.

**Table 4:** Correlational analysis of study variables.

Variable	1	2	3	4	5	6
**1** Perpetration	1					
**2** Experience	.658 *** n = 978	1				
**3** Sexual Double Standard	−.029 *n* = 984	−.113 *** n = 980	1			
**4** Sexual Permissiveness	.295 *** n = 984	.221 *** n = 980	−.112 *** n = 1003	1		
**5** Lifetime Pornography Consumption	.307 *** n = 945	.208 *** n = 941	−.044 *n* = 955	.339 *** n = 955	1	
**6** Frequency of Pornography Consumption	.355 *** n = 956	.200 *** n = 952	.019 *n* = 966	.394 *** n = 966	.525 *** n = 937	1

Note: “Prefer not to answer” responses are excluded, and only substantive responses (yes/no) are considered in the analysis. *** *p* ≤ .001.

**Table 5:** Linear regression models predicting sexual permissiveness by type of engagement in violent sexual practices.

Variable	Perpetration of Sexually Violent Practices		Experience of Sexually Violent Practices	
	*b*	95% *CI*	*b*	95% *CI*
Lifetime pornography consumption	0.60 ***	[0.47, 0.74]	0.13 ***	[0.09, 0.17]
Frequency of pornography consumption in last 12 months	0.16 ***	[0.13, 0.19]	0.16 ***	[0.13, 0.19]

Note: Regression models controlled for following variables: gender, age, education, political orientation, and sexual orientation. *b* = unstandardized regression coefficient; *CI* = confidence interval. *** *p* ≤ .001.

**Table 6:** Linear regression models predicting engagement in violent sexual practices (including % of total effect mediated).

Variable	Perpetration of Sexually Violent Practices		Experience of Sexually Violent Practices	
	*b*	95% *CI*	*b*	95% *CI*
Lifetime pornography consumption	0.50 ***	[0.34, 0.65]	0.60 ***	[0.42, 0.76]
% of total effect mediated	0.22	[0.18, 0.29]	0.21	[0.17, 0.27]
Frequency of pornography consumption in last 12 months	0.13 ***	[0.10, 0.17]	0.13 ***	[0.09, 0.17]
% of total effect mediated	0.20	[0.16, 0.24]	0.24	[0.19, 0.30]

Note: Regression models controlled for following variables: gender, age, education, political orientation, and sexual orientation. *b* = unstandardized regression coefficient; *CI* = confidence interval. *** *p* ≤ .001.

## Data Availability

The data presented in this study are available on request from the corresponding author. The data are not publicly available as they are currently being utilized for a doctoral thesis.

## Notes

1 Due to routing, some participants received fewer items.

2 Respondents who had never engaged in sexual relationships were coded as not having perpetrated any violent sexual practices during sexual encounters.

3 Respondents who had never engaged in sexual relationships were coded as not having experienced any violent sexual practices during sexual encounters.

## Appendix A

**Table A1:** Comparing the sample with the populations of Castilla-La Mancha and Spain.

Variable	Sample (*n* = 1003)	Population of Castilla-La Mancha (*n* = 2,103,588)	Population of Spain (*N* = 48,692,804)
**Gender**			
Men	50.7%	50.2%	49.1%
Women	49.3%	49.8%	50.9%
**Age in years ***	45.5	43.8	44.2
16–24 years old	10.4%	11.6%	11.2%
25–44 years old	37.5%	32.2%	29.1%
45–65 years old	43.0%	37.1%	36.9%
66+ years old	9.2%	19.0%	22.8%
**Education**			
School graduate or less	16.6%	19.3%	14.5%
High school or technical school	41.6%	54.8%	58.4%
College education	41.8%	25.9%	27.1%
**Political orientation *** (0 Left–10 Right)	5.3	5.2	4.9
**Sexual orientation**			
Heterosexual	86.6%	90.9%	93.8%
Non-heterosexual	12.4%	9.1%	6.2%

Note: The benchmark data are sourced from the Centro de Investigaciones Sociológicas (CIS) (October 2024 Barometer and the 2023 Survey on Social and Affective Relationships Post-Pandemic) and the Instituto Nacional de Estadística (INE) (1 October 2024). * The data represent means.

## Appendix B

The pretest questionnaire was conducted in two consecutive phases. During the first phase, expert reviews were carried out to identify potential issues that could introduce sources of error and/or negatively impact the experience of the respondents. In the second phase, online cognitive interviews were conducted with a convenience sample of the general population, to assess the suitability of the terminology used throughout the questionnaire.

### Appendix B.1. Expert Reviews

A panel of four Spanish-speaking experts from different universities was invited to participate in this phase of the questionnaire pretest (see Table A2). These experts were selected based on their expertise in various areas, including the potential effects of consuming pornography, and survey methodology. They received an email, at their institutional email addresses, containing detailed information about the study and an invitation letter. Along with the questionnaire, they were provided with a standardized form to indicate potential issues with the questions and/or response options. The experts conducted their reviews independently, without knowing the identities of the other reviewers. Two follow-up reminders were sent to those who did not respond before the deadline. The pretest took place between 11 and 25 December 2023, and 80% of the invited experts completed the review. Amazon gift vouchers (€50) were provided as compensation for participation.

**Table A2:** Characteristics of the expert reviewers.

Expert	Affiliation	Research Expertise	Participation
1	International University of La Rioja	Pornography consumption	Yes
2	International University of La Rioja	Pornography consumption	Yes
3	University of the Balearic Island	Pornography consumption	No
4	University of Navarra	Survey methodology	Yes

### Appendix B.2. Online Cognitive Interviews

Cognitive interviews were conducted via video calls to evaluate the appropriateness of the terminology used in the questionnaire. Participants were recruited using a convenience sampling method. Five individuals took part in this second phase of the pretest, during which think-aloud protocols were employed (see Table A3). These protocols enabled us to capture participants’ thought processes by allowing them to verbalize their thoughts while responding to the questions. We also posed specific questions (probes) regarding the understanding of the terms used in the questionnaire. An email was sent to the participants, inviting them to participate in the pretest. This email contained information about the pretest and a request for informed consent. The cognitive interviews were conducted in Spanish, via Microsoft Teams, from 2 to 4 January 2024. Each interview lasted approximately 15 min, and participants were compensated with Amazon gift vouchers (€10) for their time. The participation rate was 100%.

**Table A3:** Sociodemographic characteristics of the participants.

Gender	Age
Woman	71 years old
Woman	59 years old
Man	37 years old
Man	27 years old
Woman	17 years old

## Appendix C

**Table A4:** Linear regression models predicting sexual permissiveness by type of engagement in violent sexual practices and gender.

Variable	Men				Women			
	Perpetration of Sexually Violent Practices		Experience of Sexually Violent Practices		Perpetration of Sexually Violent Practices		Experience of Sexually Violent Practices	
	*b*	95% *CI*	*b*	95% *CI*	*b*	95% *CI*	*b*	95% *CI*
Lifetime pornography consumption	0.81 ***	[0.56, 1.06]	0.68 ***	[0.45, 0.91]	0.49 ***	[0.34, 0.64]	0.49 ***	[0.34, 0.64]
Frequency of pornography consumption in last 12 months	0.17 ***	[0.13, 0.21]	0.17 ***	[0.13, 0.20]	0.14 ***	[0.09, 0.20]	0.14 ***	[0.09, 0.20]

Note: Regression models controlled for the following variables: gender, age, education, political orientation, and sexual orientation. *b* = unstandardized regression coefficients; *CI* = confidence interval. *** *p* ≤ .001.

**Table A5:** Linear regression models predicting engagement in violent sexual practices by gender (including % of total effect mediated).

Variable	Men				Women			
	Perpetration of Sexually Violent Practices		Experience of Sexually Violent Practices		Perpetration of Sexually Violent Practices		Experience of Sexually Violent Practices	
	*b*	95% *CI*	*b*	95% *CI*	*b*	95% *CI*	*b*	95% *CI*
Lifetime pornography consumption	0.56 ***	[0.25, 0.86]	0.35 *	[0.07, 0.62]	0.48 ***	[0.31, 0.64]	0.68 ***	[0.45, 0.91]
% of total effect mediated	0.29	[0.21, 0.47]	0.37	[0.24, 0.71]	0.14	[0.10, 0.19]	0.17	[0.13, 0.23]
Frequency of pornography consumption in last 12 months	0.10 ***	[0.05, 0.15]	0.08 ***	[0.03, 0.12]	0.20 ***	[0.15, 0.26]	0.29 ***	[0.21, 0.37]
% of total effect mediated	0.29	[0.21, 0.42]	0.31	[0.22, 0.48]	0.10	[0.08, 0.13]	0.13	[0.10, 0.16]

Note: Regression models controlled for the following variables: gender, age, education, political orientation, and sexual orientation. *b* = unstandardized regression coefficients; *CI* = confidence interval. * *p* ≤ .05, *** *p* ≤ .001.
